# Human Intracranial High Frequency Oscillations (HFOs) Detected by Automatic Time-Frequency Analysis

**DOI:** 10.1371/journal.pone.0094381

**Published:** 2014-04-10

**Authors:** Sergey Burnos, Peter Hilfiker, Oguzkan Sürücü, Felix Scholkmann, Niklaus Krayenbühl, Thomas Grunwald, Johannes Sarnthein

**Affiliations:** 1 Neurosurgery Department, University Hospital Zurich, Zurich, Switzerland; 2 Institute of Neuroinformatics, ETH Zurich, Zurich, Switzerland; 3 Swiss Epilepsy Centre, Zurich, Switzerland; 4 Biomedical Optics Research Laboratory, Neonatology Department, University Hospital Zurich, Zurich, Switzerland; 5 Neurology Department, University Hospital Zurich, Zurich, Switzerland; 6 Center for Integrative Human Physiology, University of Zurich, Zurich, Switzerland; University Paris 6, France

## Abstract

**Objectives:**

High frequency oscillations (HFOs) have been proposed as a new biomarker for epileptogenic tissue. The exact characteristics of clinically relevant HFOs and their detection are still to be defined.

**Methods:**

We propose a new method for HFO detection, which we have applied to six patient iEEGs. In a first stage, events of interest (EoIs) in the iEEG were defined by thresholds of energy and duration. To recognize HFOs among the EoIs, in a second stage the iEEG was Stockwell-transformed into the time-frequency domain, and the instantaneous power spectrum was parameterized. The parameters were optimized for HFO detection in patient 1 and tested in patients 2–5. Channels were ranked by HFO rate and those with rate above half maximum constituted the HFO area. The seizure onset zone (SOZ) served as gold standard.

**Results:**

The detector distinguished HFOs from artifacts and other EEG activity such as interictal epileptiform spikes. Computation took few minutes. We found HFOs with relevant power at frequencies also below the 80–500 Hz band, which is conventionally associated with HFOs. The HFO area overlapped with the SOZ with good specificity > 90% for five patients and one patient was re-operated. The performance of the detector was compared to two well-known detectors.

**Conclusions:**

Compared to methods detecting energy changes in filtered signals, our second stage - analysis in the time-frequency domain - discards spurious detections caused by artifacts or sharp epileptic activity and improves the detection of HFOs. The fast computation and reasonable accuracy hold promise for the diagnostic value of the detector.

## Introduction

For patients with epilepsy refractory to medication, neurosurgical resection of the epileptogenic zone is a therapeutic option. As the gold standard in current practice, the epileptogenic zone is approximated by the seizure onset zone (SOZ) defined by presurgical analysis of intracranial EEG (iEEG) [1]. Over the last years, High Frequency Oscillations (HFOs) [2] have been evaluated as a new biomarker for the epileptogenic zone [3]–[10]. HFOs are defined as spontaneous EEG patterns in the frequency range of 80–500 Hz, consisting of at least four cycles that can be “clearly” distinguished from background noise [2]. HFOs can be recorded during the interictal period and thereby potentially reduce recording time, discomfort and risk for patients. This definition comprises events like ripples (80–250 Hz) and fast ripples (FRs) (250–500 Hz) [8], but the characteristics of clinically relevant HFOs have not yet been agreed upon.

Since visual marking of HFOs is highly time-consuming, several algorithms for automatic or semi-automatic detection of HFOs have been proposed [11]–[19]. While earlier detectors rely rather on thresholds in the time domain, a number of recent detectors also incorporate the frequency domain, which is computationally more demanding.

We propose here the detection of Events of Interest (EoIs) in a first stage of analysis, followed by a second stage that selects HFOs from the set of EoIs. As an extension of the detectors proposed by [14], [20], we analyze the instantaneous power spectral density of an EoI. In order to obtain a precise instantaneous power spectral density, we use the Stockwell transform [21] that enables an excellent time-frequency decomposition of a signal. The Stockwell transform can be regarded as an generalization of the short-time Fourier transform and a further development of the continuous wavelet transform, combining both advantages [22]. Within this signal-processing framework, we can accurately distinguish HFOs, which are short-lived, from long-lived low frequency activity as it may occur during interical epileptiform spikes (IES). The proposed algorithm has low computational run time and is easy to implement.

We applied the detector to intracranial presurgical diagnostic EEG recordings from six epilepsy patients. We used channels with large numbers of HFOs to identify the epileptogenic zone [3], [23], [24]. We compared this zone to the current gold standard, the SOZ as defined by the clinical presurgical workup based on ictal and interictal activity. In addition, we studied the spectral frequency of individual HFOs.

## Methods

### Patient selection

We included all patients that were implanted with intracranial electrodes at the Neurosurgery Department from March 2012 to March 2013 and where iEEG could be recorded with a sampling frequency of at least 2000 Hz. This selection criterion resulted in six patients (all male, median age 25 years). Patient characteristics and implantation sites are given in Table 1.

**Table 1:** Clinical data and implantation sites.

Patient	Age	Age at onset	MRI	Histology	Number of implanted electrodes	Implantation sites	Procedure
1	21	2	HS (left MTL)	HS type 1a	8	AL, ECL, HL, PCL AR, ECR, HR, PCR	sAHE
2	21	3	HS (left MTL) & right frontal FCD	–	7	AL, ECL, HL, PH ECR, HR, PCR	DBS
3	34	25	bilateral HS	–	5	ECL, HL AR, ECR, HR	DBS
4	48	2	HS (left MTL)	HS (no classification)	8	AL, ECL, HL, PCL AR, ECR, HR, PCR	sAHE
5	27	1	right frontal FCD	FCD type IIa	3	FAR, TFR, FPR	LE
6	22	11	no lesion	–	5	TLL, TBAL, TBPL TBAL, TBPR	–

Pathologies: FCD focal cortical dysplasia; HS hippocampal sclerosis. Procedures: DBS deep brain stimulation; LE extended lesionectomy; sAHE selective amygdala-hippocampectomy. Implantation sites: AL amygdala left; AR amygdala right; EL entorhinal cortex left; ER entorhinal cortex right; FAR frontal anterior right; FL frontal lobe; FPR frontal posterior right; HL hippocampus left; HR hippocampus right; MTL mesial temporal lobe; PL perirhinal cortex left; PR perirhinal cortex right; TBAL temporal basal anterior left; TBAR temporal basal anterior right; TBPL temporal basal posterior left; TBPR temporal basal posterior right; TR depth frontal right; TLL temporal lateral left.

### Ethics statement

Collection of personal patient data and retrospective scientific workup was approved by the institutional ethics review board (Kantonale Ethikkommission KEK-ZH-Nr. 2012-0212) and collection of patients' written informed consent was waived.

### Electrode types and implantation sites

Intracranial depth macro electrodes and subdural strips and grids were implanted at locations planned according to the results of the previous non-invasive presurgical workup.

Mesiotemporal depth electrodes (1.3 mm diameter, 8 contacts of 1.6 mm length, spacing between contacts 5 mm, ADTech, www.adtechmedical.com) were implanted stereotactically into the amygdala, the hippocampal head and the entorhinal and perirhinal cortex bilaterally.

For cortical sites, a combination of depth and subdural strip and grid electrodes (contact diameter 4 mm with a 2.3 mm exposure, spacing between contact centers 10 mm) was placed after craniotomy. Here depth electrodes were implanted into the supposed center of bottom-of-sulcus dysplasias as defined be morphometric postprocessing of MR images [25].

An image-guidance system was used (StealthStation, Medtronic, www.medtronic.com). Pre- and post-implantation magnetic-resonance imaging (MRI) and computer tomography (CT) scans were used to locate each contact anatomically along the electrode trajectory.

### Data acquisition

Data was recorded for presurgical evaluation starting from the day after electrode implantation. The recording was performed in the intensive monitoring unit under video surveillance at the Swiss Epilepsy Centre. Intracranial data was acquired at 4000 Hz with an ATLAS recording system (Neuralynx, www.neuralynx.com) and downsampled to 2000 Hz for HFO analysis. In addition, surface EEG and the submental electromyogram (EMG) were recorded. Intracranial data was recorded against a common reference and then transformed to a bipolar montage for further analysis.

### Data selection

We selected interictal samples of five minutes of slow-wave sleep because of reduced muscle activity and because HFOs occur more often during slow-wave sleep than during wakefulness [7], [26]. Sleep staging was performed based on scalp EEG, electrooculogram (EOG), EMG and video recordings. The selected data samples were separated from epileptic seizures by at least three hours to reduce the influence of seizures on our analysis.

### Data analysis

**Figure 1:**
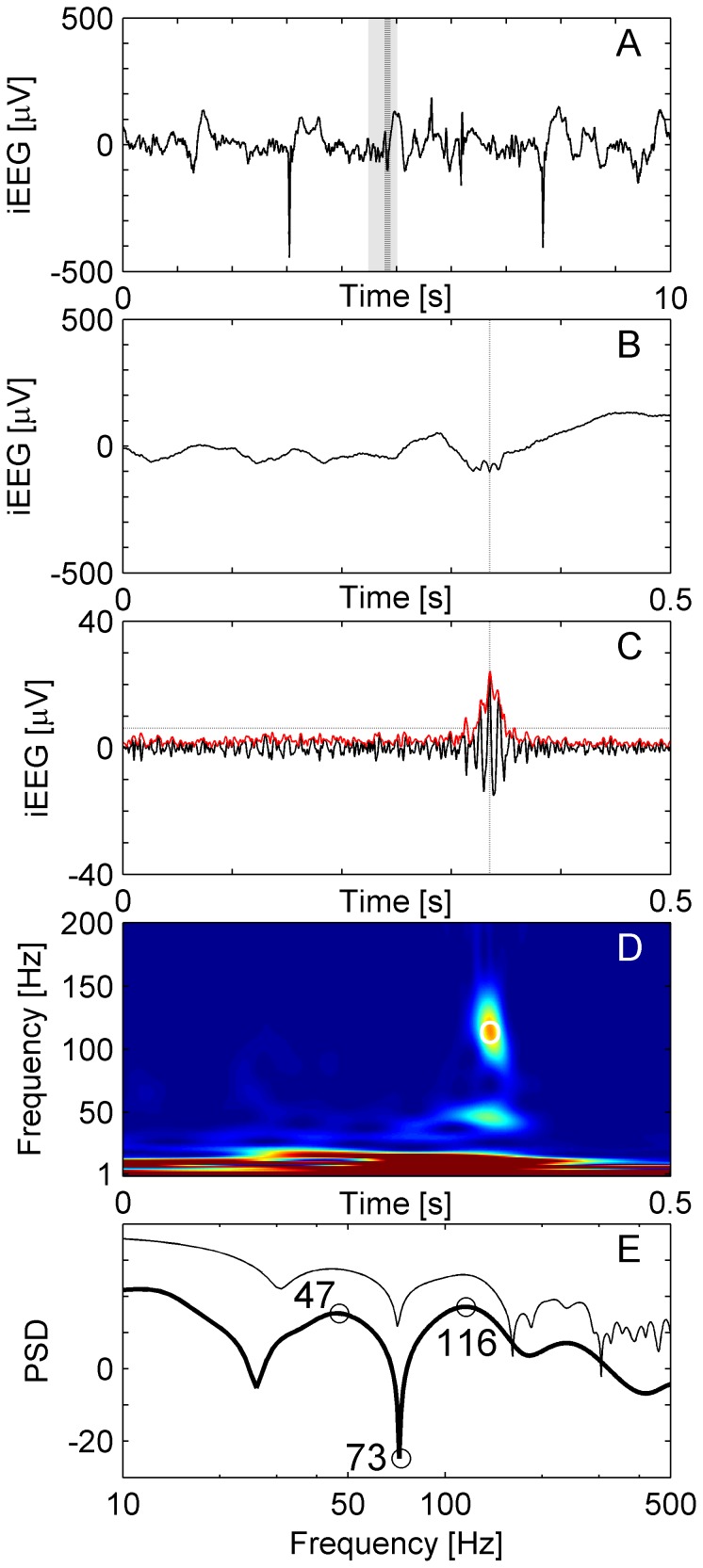
HFO in a temporomesial recording. (**A**) Raw iEEG 10 s epoch from channel HL1 in patient 1. (**B**) Raw iEEG at extended time scale of 500 ms. (**C**) Filtered iEEG of panel B with envelope (red line). The envelope satisfies the criteria for an EoI (Stage 1 of detection). The peak of the envelope is marked by dashed vertical lines in panels A, B and C. (**D**) Time frequency representation of the iEEG of panel B. The circle marks the peak of the envelope of the EoI. The “blob” represents the HFO. (**E**) Power spectral density (PSD, unit: 10log_10_μV^2^Hz^−1^) at the peak of the EoI. For the event illustrated here, there is a HFO peak at 116 Hz, a trough at 73 Hz and a low frequency peak at 47 Hz. The thin line shows the PSD of the same data calculated by the short-time Fast Fourier Transform for comparison.

The aim of our detector was to distinguish HFOs from other iEEG activity and artifacts. In this paragraph, we present the rationale of the detector and we present details in the following sections. Our HFO detector involves two stages. In the first stage, after pre-filtering the signal, possible events of interest (EoIs) were detected based on thresholds in the time domain. This signal processing stage was optimized to ensure a high sensitivity and we accepted a low specificity to obtain a large number of EoIs. In the second stage, we reviewed all EoIs in the time-frequency domain in order to recognize HFOs. Following the recommendation in [23], this was first done by visual inspection in separate windows as in Figure 1 for up to 10 channels simultaneously. In addition, both stages of HFO detection were performed automatically with custom scripts written in MATLAB (www.mathworks.com).

### Stage 1: Detection of EoIs

First, the signal was band-pass filtered from 80 to 500 Hz. We used an Infinite Impulse Response (IIR) Cauer filter, 60 dB minimum lower/upper stop band attenuation, 0.5 dB maximum pass band ripple, 10 Hz lower/upper transition width, forward and reverse filtered in order to avoid phase distortion. To ensure filter stability we performed extensive testing with the MATLAB filter design toolbox. While other groups use FIR filters [11], [12], [27], we prefer IIR filters because IIR filters reduce computational run time 100-fold in our algorithm and have a sharp cut-off as well as a narrow transition width. Figure 1 shows a typical raw signal (panels A and B), which was then band-passed (panel C).

We then scanned the signal for events of high amplitude and sufficient duration to qualify as EoIs. The detection of EoIs proceeded in the following steps [11].

1. We calculated an envelope of the band-passed signal using the Hilbert transformation [12], [28].
2. We calculated the standard deviation (SD) of the envelope of the 5 min of data. We then set a threshold at the mean of the envelope plus 3 SD.
3. An event was marked when the envelope exceeded the threshold. The duration of the event was defined as the interval between upward and downward crossing of 0.5*threshold. If its duration exceeded 6 ms, this event qualified as an EoI.
4. We merged EoIs with an inter-event-interval of less than 10 ms into one EoI.
5. Events not having a minimum of 6 peaks (band-passed signal rectified above 0 μV) greater than 2 SD from the mean baseline signal were rejected [11].

### Stage 2: Recognition of HFOs among EoIs

In the second stage, we distinguished HFOs from EoIs that were elicited by other EEG activity and artifacts [29], [30]. We implicitly assume that a HFO appears as a short-lived event with an isolated spectral peak at a distinct frequency [12], [20]. We therefore reviewed all EoIs and transformed the period of [−0.5 s, +0.5 s] into time-frequency space.

For this transformation we used the Stockwell transform [21] because it yields superior peak sharpness compared to the short-time Fourier Transform at similar computational speed for our time series of 1 s. In both cases, the frequency resolution is 1 Hz. Compared to the short-time Fourier transform, the Stockwell transform does not use a window with a constant width but relies on a frequency dependent window width, which increases the time-frequency resolution. Thus, the Stockwell transform is a hybrid of the short-time Fourier transform and the continuous wavelet transform, combining the advantages of both methods [21], [31], [32].

Figure 1D shows an EoI in time-frequency space. Only a period of 0.5 s around the EoI is displayed to mask boundary effects. To qualify as a HFO, the EoI must exhibit a high frequency peak, which is isolated from low frequency activity by a spectral trough. To recognize HFOs automatically, we analyzed the instantaneous power spectra of the TF representation (Figure 1E, PSD unit: 10log_10_μV^2^Hz^−1^) around the maximum of the envelope (vertical dashed line in Figure 1C). The instantaneous power spectra were computed for all time points of the envelope within the full width at half maximum above the threshold (FWHM). This boundary assures that the maximum of the envelope and its neighborhood above the threshold is taken into analysis. The instantaneous power spectrum for each time point was parameterized by three frequency bins in the following way.

1. We selected the high frequency peak (HiFP) as the spectral peak of a putative HFO. This HiFP was selected in the spectral range from f_min_ (HiFP)  =  60 Hz to 500 Hz. The lower edge (60 Hz) was chosen heuristically to avoid 50 Hz line hum in the signal.
2. We defined the trough as the minimum in the range between f_min_ (trough)  =  40 Hz and the HiFP.
3. We defined the low frequency peak (LoFP) as the closest local maximum below the trough.

These three frequency bins were used to distinguish HFOs in the instantaneous spectrum at each time point within the FWHM. To qualify as a HFO, we demand a trough of sufficient depth Power(Trough)/Power(HiFP) < 0.8 and a HiFP peak of sufficient height Power(HiFP)/Power(LoFP) > R_thr_  =  0.5. These two conditions have to be satisfied by all instantaneous power spectra within the FWHM.

For further analysis, we defined the high frequency peak, the trough and the low frequency peak of an individual HFO as the HiFP, trough and LoFP at the time point when the envelope reaches its peak.

**Figure 2:**
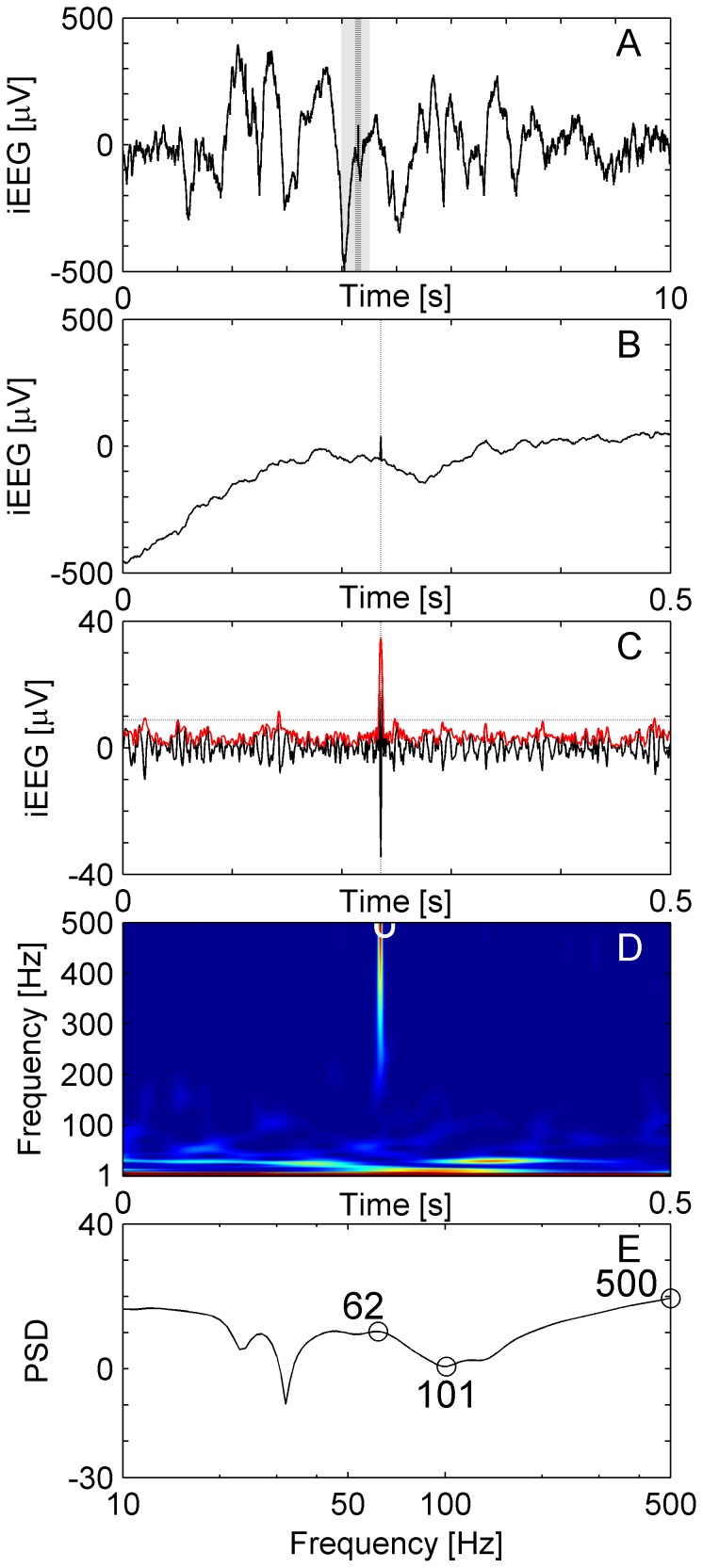
Sharp artifact. (**A**) Raw iEEG data 10 s epoch from a frontal channel in patient 6. (**B**) Raw iEEG at extended time scale. (**C**) Filtered data (blue line) with envelope (red line). The envelope satisfies the criteria for an EoI (Stage 1 of detection). While the high-frequency activity is separated by a trough (**D, E**), it is excluded from acceptance as HFO because the peak of the spectral power appears at frequencies above 500 Hz.

Figure 2 shows a short sharp artifact, which qualified as an EoI in Stage 1 of the analysis. It was excluded from acceptance as a HFO because the peak of the spectral power appeared at frequencies above 500 Hz (Figure 2E).

### Definition of the SOZ

In all patients presented here, the surgical planning of the resection area was based on the SOZ, which we take as the gold standard. In conventional analysis of clinical EEG recordings, epileptic seizures are reviewed visually. The channels with the earliest signs of ictal EEG activity define the SOZ [33]. It is usually necessary though not sufficient to resect the SOZ as part of the epileptogenic zone, which in turn is defined as the area that has to be resected to achieve seizure freedom. In most cases, the epileptogenic zone also includes the epileptogenic lesion (if present and removable) and adjacent regions of consistent early spread of seizure activity. In addition, epileptiform potentials like IES or “spike-waves” are reviewed to delineate irritative zones of cortex against healthy tissue.

### Separate data sets for training and testing the detector

To optimize parameters for HFO detection, we selected the data from patient 1 as the training set. In this patient, the SOZ contained only two channels (HL1-HL2, HL2-HL3; Table 2). We assumed that these channels indicate the epileptogenic zone [1] and therefore optimized parameters for detection of these channels. To test the performance of the detector, we selected patients 2–6 as the test set.

**Table 2:** Spatial HFO distribution compared to seizure onset zone (SOZ).

Patient	HFO area [number of channels]	HFO area [% of all channels]	Channels within HFO area	Seizure onset zone (SOZ)	TP	TN	FP	FN	Sens	Spec
1	6	11%	HL 1, 6; PL 1, 2; ER 1, 2	HL 1, 2	1	49	5	1	50%	91%
2	3	60%	HL 7; HR 2; ER 7	HL 1; EL 1; HR 1, 2, 3; ER 1, 2	1	40	2	6	14%	95%
3	5	14%	HL 1; HR 1, 2; AR 1, 2	HL 1, 2; AR 1, 2	3	29	2	1	75%	94%
4	6	11%	EL 1, 2; PL 1; HL 1; PR 1; AR 1	EL 1,2; HL 1,2	3	49	3	1	75%	94%
5	29	74%	FPR 2, 3, 4, 5, 6, 8, 9, 10, 11, 12, 13, 14, 15; FAR 4, 5, 6, 7, 10, 11, 12, 13, 14; TR 1, 2, 3, 4, 5, 6, 7	FPR 1, 2, 3, 4, 9, 10, 11, 12; FAR 5, 6, 13, 14; TR 1, 2, 3, 4	15	9	14	1	94%	39%
6	2	6%	TLL 28, 29	TLL 18, 19, 20, 21, 22, 23, 26, 27, 28, 29, 30, 31 TBAL 1, 2, 3	2	24	0	13	13%	100%

Channels with high number of HFOs define the HFO area with a threshold determined by a half maximum. HFO areas and SOZ show partial overlap in all 6 patients. Channel HL1 indicates a bipolar recording from contacts HL2-HL1. For abbreviations of electrode names, see Table 1.

### Definition of the HFO area

We computed the rates of automatically detected HFOs in the 5 min data sample. We analyzed the spatial distribution of HFOs rates for each patient by ranking channels by their HFO rate. The width of distribution was captured by its half maximum. The channels with HFO rate higher than the half maximum constitute the HFO area, which we assume to indicate the location of the epileptogenic zone.

In addition to the half maximum threshold, we also tried Kittler's method to separate channels with high HFO rates [34].The results for sensitivity and specificity were similar to within a few percent points so that the data are not shown here.

### Statistical analysis

The channels within the HFO area were counted as true positives (TP) if they were located within the SOZ and were counted as false positives (FP) if they were located outside of the SOZ. The channels outside of the HFO area were counted as false negatives (FN) if they were located within the SOZ and were counted as true negatives (TN) if they were located outside of the SOZ. The sensitivity was calculated as sens  =  TP/(TP + FN), the false-positive rate as FPR  =  FP/(FP + TN) and the specificity as spec  =  1 – FPR. 95% confidence intervals (CI) were estimated based on the binomial distribution. We use the values of the sensitivity and the specificity to quantify the performance of our detector in the sense of how strongly the HFO area overlaps with the SOZ.

## Results

### Examples of EoIs and HFOs in individual patients

Figure 1 shows an example of a HFO recorded from the hippocampal channel HL1 of patient 1. The HFO appears as a short-lived event that is clearly distinct from low-frequency activity (Figure 1D).

**Figure 3:**
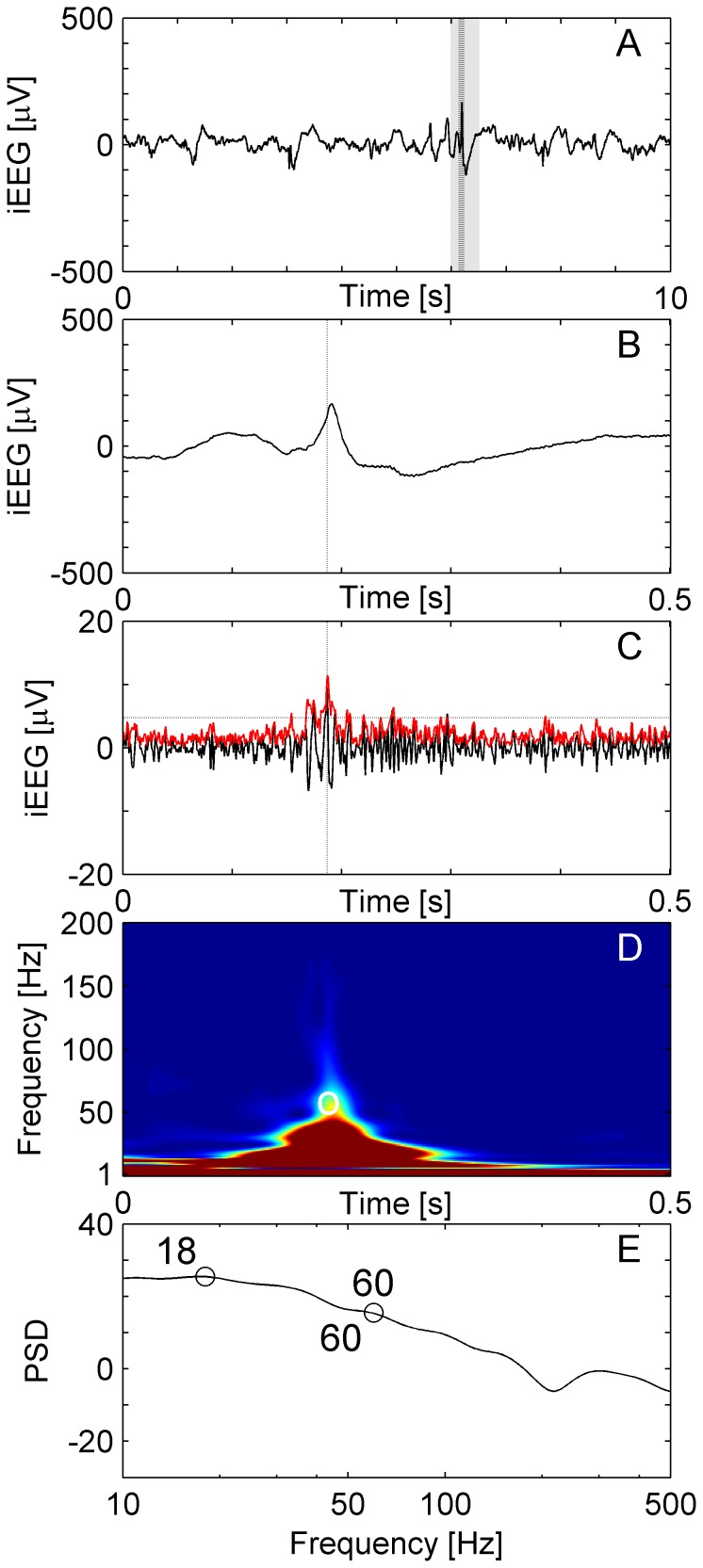
Interictal Epileptiform Spike (IES) without HFO. (**A**) Raw iEEG 10 s epoch from channel HL6 in patient 1. (**B**) Raw iEEG at extended time scale of 500 ms. (**C**) Filtered iEEG of panel B with envelope (red line). The envelope satisfies the criteria for an EoI (Stage 1 of detection). (**D, E**) The peak of the high-frequency activity and the trough coincide at 60 Hz, i.e. the EoI is not separated from low-frequency activity by a trough and the EoI is therefore excluded from acceptance as HFO.

The IES shown in Figure 3 also appears as a short-lived event in the band-passed channel (Figure 3C) and qualifies as an EoI in Stage 1 of our analysis. The EoI is not accepted as a HFO in Stage 2 of the analysis because the event shows a smooth transition between high and low frequency activity in the absence of a trough.

**Figure 4:**
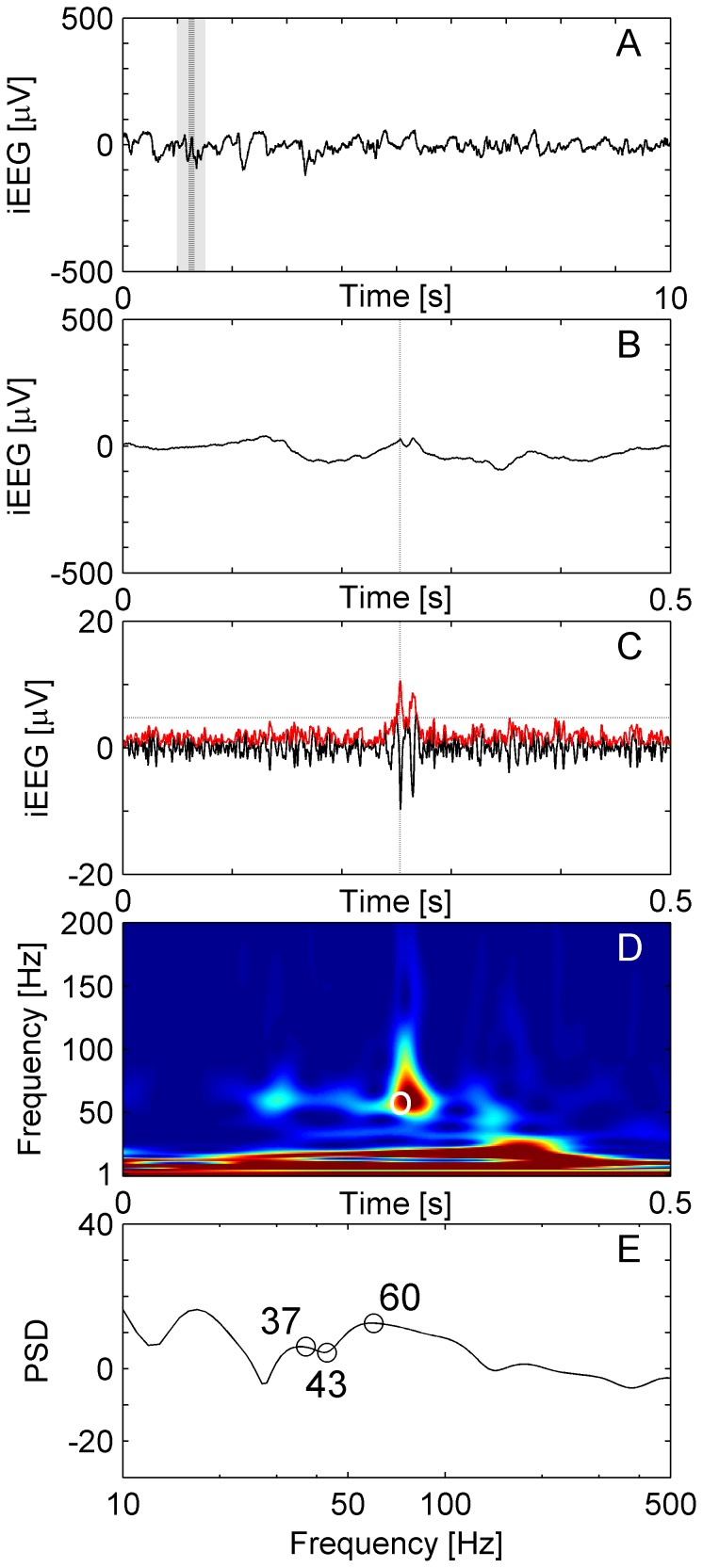
HFO with frequency peak below 80 Hz. (**A**) Raw iEEG data 10 s epoch from channel HL6 in patient 1. (**B**) Raw iEEG at extended time scale of 500 ms. (**C**) Filtered iEEG with envelope (red line). The envelope satisfies the criteria for an EoI (Stage 1 of detection). The EoI is salient enough to be separated from low-frequency activity by a trough (**D, E**). This EoI has the visual appearance of a HFO but the peak frequency is around 60 Hz. Therefore, in Stage 2 the lowest boundary for a HiFP was chosen at 60 Hz and this EoI was accepted as HFO by our detector.

Figure 4 depicts an EoI that appeared as a HFO in visual inspection. Energy and duration of the event met the Stage 1 requirements of an EoI. However, its high frequency peak is at 60 Hz, which is lower than common definition of HFOs (>80 Hz). Therefore, in the Stage 2, we limited the lowest boundary for HiFP at 60 Hz and this EoI was accepted as a HFO by our detector.

**Figure 5:**
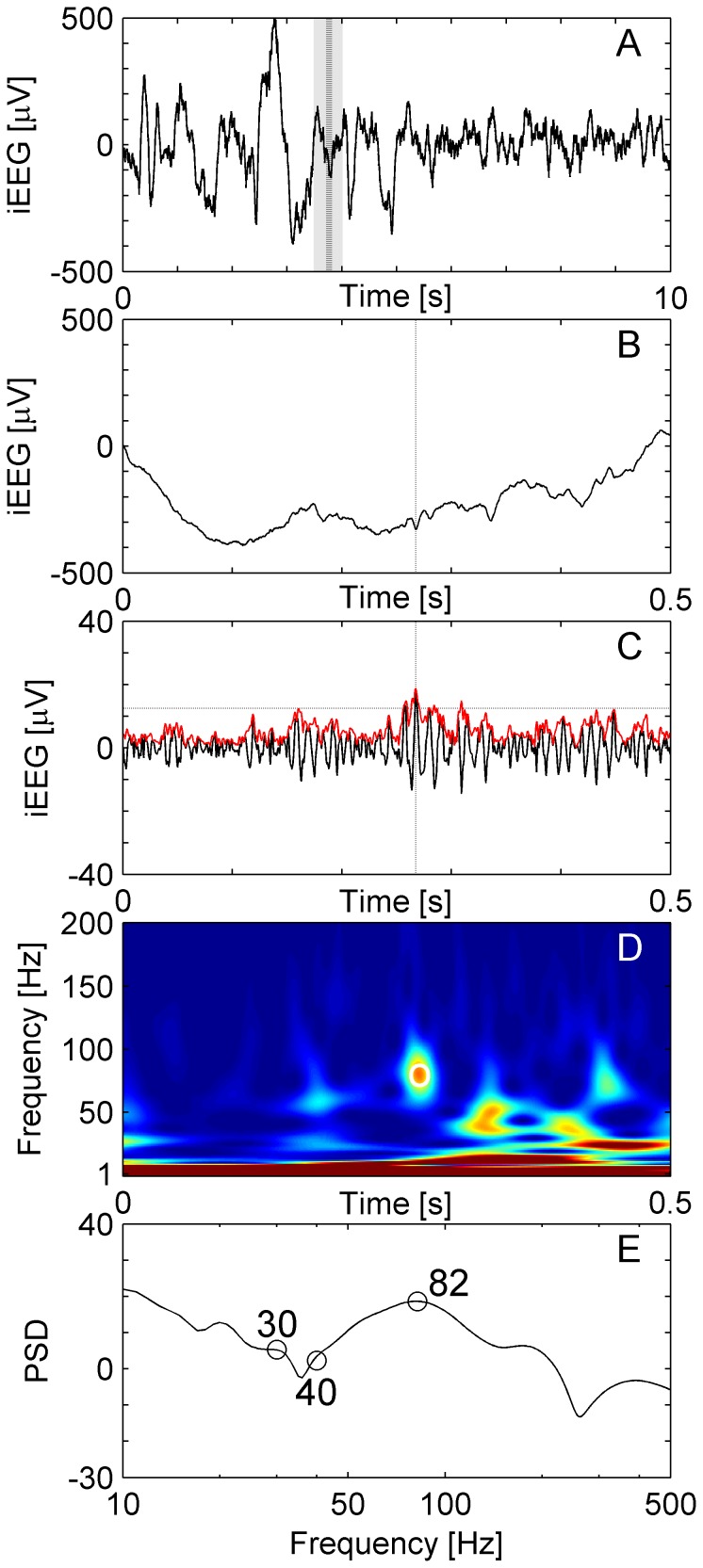
HFO in a neocortical recording. (**A**) Raw iEEG data 10 s epoch recorded from a frontal channel in patient 6. (**B**) Raw iEEG at extended time scale of 500 ms. (**C**) Filtered data with envelope (red line). The envelope satisfies the criteria for an EoI. The peak of the envelope is marked by dashed vertical lines in panels A, B and C. (**D**) Time frequency representation of the iEEG of panel B. The circle marks the peak of the envelope of the EoI. (**E**) Power spectral density (PSD, unit: 10log_10_μV^2^Hz^−1^) at the peak of the envelope. The high frequency peak is at 82 Hz, the trough at 40 Hz and the low frequency peak at 30 Hz for this event, which was accepted as HFO in Stage 2 of the detection.

A HFO from a neocortical recording in patient 6 is shown in Figure 5. The HFO appears less salient than in temporomesial recordings.

### Characteristics of HFOs in individual patients

Table 3 presents the total number of EoIs and HFOs that were detected in each individual patient. The mean characteristics of HFOs are all in the same range for both temporomesial implantation sites (patients 1, 2, 3 and 4) and neocortical implantation sites (patients 5 and 6). Among all EoIs detected in stage 1, an average 66% of events were accepted as HFOs. Patient 4 showed the lowest acceptance rate 32%, and patient 5 and 6 had the highest rates 83% and 79% respectively. Furthermore, our algorithm allowed us to detect HFOs with widely varying duration (Table 3).

**Table 3:** Temporal and spectral characteristics of HFOs in individual patients.

Patient	Number of channels	Number of EoIs	Number of HFOs	Acceptance rate [%]	Number of ripples/fast ripples # ripple/# FR	HFO rate per channel, mean±SD [1/min]	HFO duration, mean±SD [ms]	HFO peak, mean±SD [Hz]	Trough frequency, mean±SD [Hz]	Low frequency peak, mean±SD [Hz]	Amplitude, mean±SD [μV]
1	56	796	378	47%	337/41	7 ± 11	58 ± 34	113 ± 55	58 ± 20	39 ± 18	16 ± 26
2	49	245	91	37%	77/14	2 ± 4	47 ± 26	141 ± 78	57 ± 19	39 ± 17	77 ± 114
3	35	882	387	44%	292/95	11 ± 23	41 ± 15	159 ± 90	71 ± 33	42 ± 26	57 ± 40
4	56	1013	323	32%	317/6	1 ± 9	58 ± 25	93 ± 30	56 ± 16	38 ± 13	20 ± 22
5	39	3724	3104	83%	3104/0	80 ± 29	91 ± 48	94 ± 16	52 ± 11	37 ± 12	82 ± 61
6	36	576	457	79%	457/0	13 ± 19	67 ± 48	82 ± 16	51 ± 11	37 ± 10	21 ± 11

For each patient we present the total number of EoIs detected in Stage 1 and the number of HFOs accepted in Stage 2. Based on their peak frequency, HFOs were then classified into ripples (80–200 Hz) and FRs (200–500 Hz).

**Figure 6:**
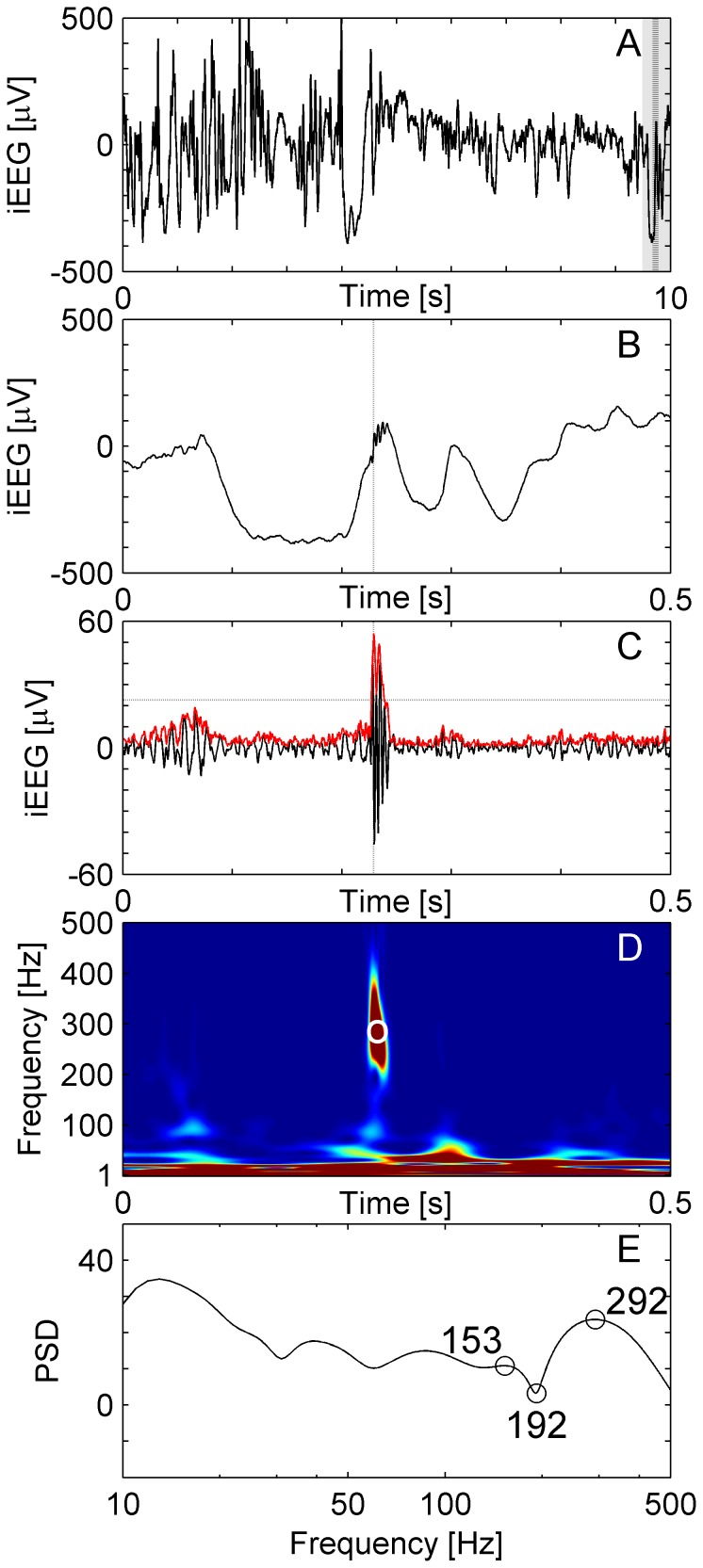
Fast ripple in a temporomesial recording. (**A**) Raw iEEG data 10 s epoch recorded from channel AR1 in patient 3. (**B**) Raw iEEG at extended time scale of 500 ms. (**C**) Filtered data with envelope (red line). The envelope satisfies the criteria for an EoI. The peak of the envelope is marked by dashed vertical lines in panels A, B and C. (**D**) Time frequency representation of the iEEG of panel B. The circle marks the peak of the envelope of the EoI. (**E**) Power spectral density (PSD, unit: 10log_10_μV^2^Hz^−1^) at the peak of the envelope. The high frequency peak is at 292 Hz, the trough at 192 Hz and the low frequency peak at 153 Hz for this event, which was accepted as a FR in Stage 2 of the detection.

We classified HFOs by their high frequency peak into ripples (80–200 Hz) and FRs (200–500 Hz) and presented the respective rates as a column in Table 3. In our patient group, we observed a predominance of ripples over FRs. This predominance may be accentuated by our classification method, which classifies a HFO as a ripple even if a FR occurs simultaneously. Figure 6 represents an example of correctly identified FR.

**Figure 7:**
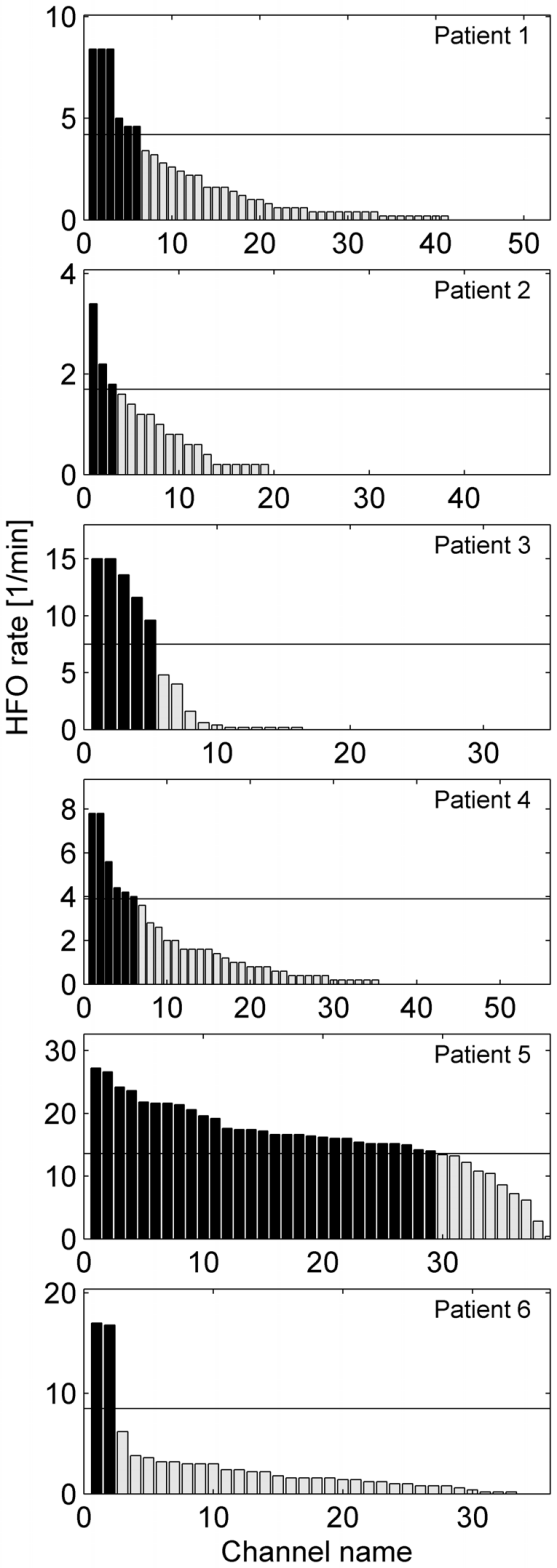
Channels ranked by HFO rate. Channels with high HFO rate are assumed to mark the epileptogenic zone. Channels with HFO rate above the half maximum (black bars) constitute the HFO area. The channel locations of the HFO area in each patient is given in Table 3.

Figure 7 shows the channel count as a function of HFO rate in each individual patient. The HFO area is given in Table 2 in terms of absolute number of channels and also normalized by the total number of channels. The spatial distributions of HFOs in patients 1, 2, 3, 4 and 6 are focused on a few channels. Widespread occurrence of HFOs as in patient 5 suggests a widespread epileptogenic zone.

**Figure 8:**
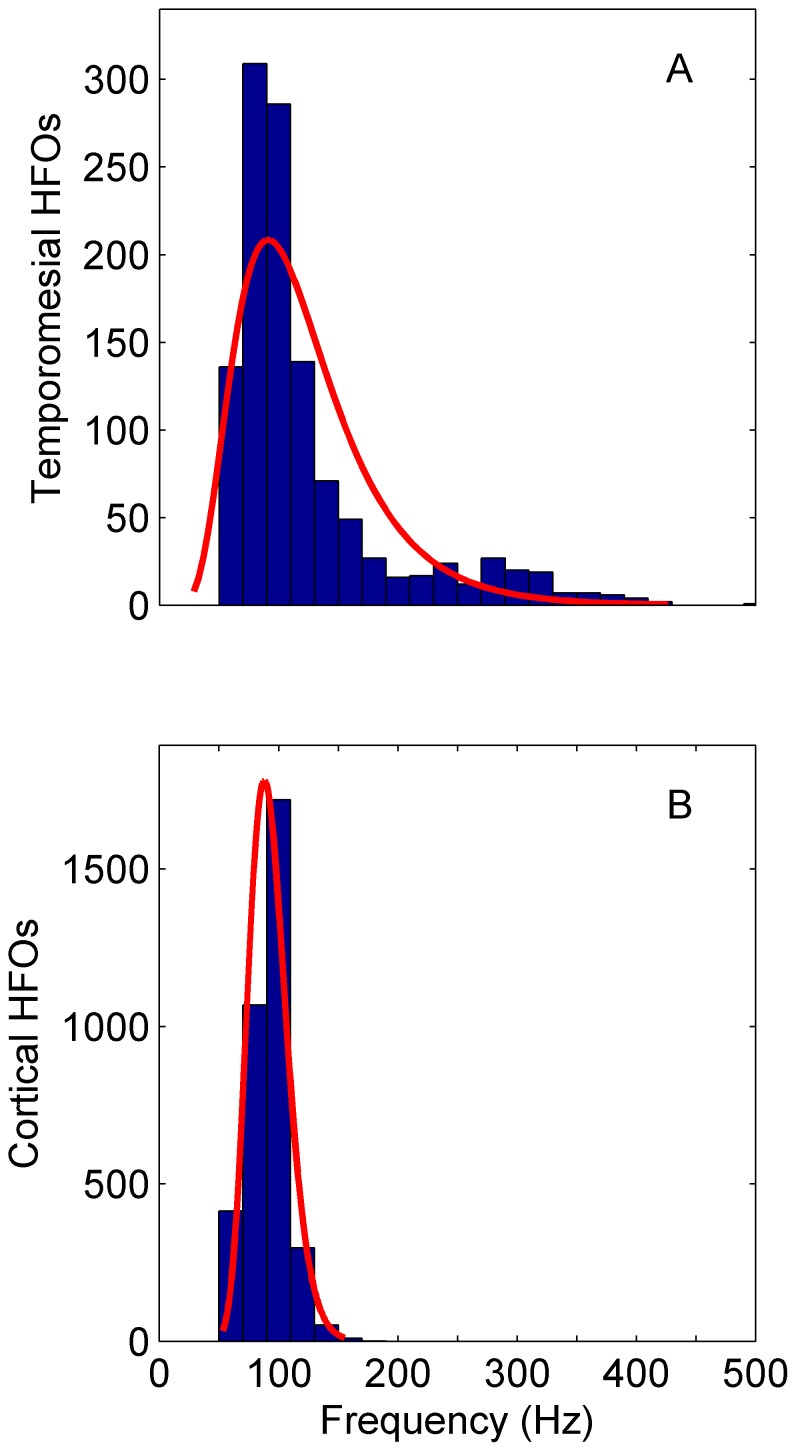
HFO peak frequency distribution. (**A**) Total of all HFOs recorded from the four patients with temporomesial electrodes (N  =  1179). The sharp edge to low frequencies stems from the frequency threshold of 60 Hz of the detector. (**B**) Total of all HFOs recorded from the two patients with neocortical electrodes (N  =  3561). The distributions are fitted with a lognormal function (red line). The frequency peaks of HFOs from temporomesial recordings are more widely distributed than those from neocortical recordings.

As an advantage of our HFO-detection in the time-frequency domain, we can determine the peak frequency for each individual HFO. Figure 8 shows the distribution of peak frequencies for medial temporal and neocortical recordings separately. The peaks of HFOs from temporomesial recordings are more widely distributed in frequency than those from neocortical recordings. Furthermore, 22% of temporomesial and also 22% of neocortical HFOs exhibit maxima below 80 Hz.

### Comparison with the clinical delineation of the SOZ

Channels with HFO rates ≥ half maximum are listed in Table 2 for all six patients. HFO channels corresponded to the location of the SOZ to a varying degree.

In patient 1, the SOZ were found in left temporomesial channels (HL1, HL2) and a left-sided selective amygdala-hippocampectomy was performed. The three channels with the highest HFO rate were also in the left hemisphere (HL1, PL1, and PL2). There were 3 channels with markedly lower HFO rates but still within the HFO area (HL6, ER1, ER2) that are counted as FP, resulting in spec  =  91% CI [80% 97%]. Channel HL2 from the SOZ showed a HFO rate below the half maximum resulting in sens  =  50% CI [13% 99%]. Visual inspection as a complement to automatic HFO detection did not reveal additional HFOs in this channel.

In patient 2, HFOs were found bilaterally in temporomesial channels in agreement with the SOZ, which was also found bilaterally. The individual channels were not the same, which results in only one TP and a low sens  =  14% CI [4% 58%]. Channels outside of the SOZ did not show HFOs, resulting in an adequate spec  =  95% CI [84% 99%]. Based on the location of the SOZ, the therapeutic decision was not to resect but to offer deep brain stimulation (DBS) to this patient. HFO analysis would have led to the same therapeutic decision.

In patient 3, the HFO area closely overlapped with the SOZ as shown by the high values of sens  =  75% CI [20% 99%] and spec  =  94% CI [79% 99%]. Again, the SOZ was bilateral so that DBS was offered also to this patient.

In patient 4, the SOZ and HFOs were found in left temporomesial channels and a left-sided selective amygdala-hippocampectomy was performed. In addition, HFOs were found in one right-sided channel, resulting in sens  =  75% CI [20% 99%] and spec  =  94% CI [84% 99%].

In patient 5, all but one channel of the SOZ were found within the HFO area resulting in sens  =  94% CI [70% 100%]. In addition, there were 14 false positive HFO channels, so that spec  =  39% CI [20% 61%]. An extended lesionectomy was performed in this patient. However, the postsurgical outcome was poor, so that the patient was operated on for a second time, which resulted in a more than 90% reduction of seizure frequency though not in complete seizure freedom. Thus, the low spec  =  39% may be a consequence of the unsuccessful delineation of the SOZ.

In patient 6, the SOZ extended over 15 of 36 channels. High HFO rates occurred in a large number of channels and an exceptionally high rate in two channels. Only these two channels constituted the HFO area, resulting in a high spec  =  100% CI [86% 100%] but a low sens  =  13% CI [2% 40%]. Surgery on this patient was postponed for clinical reasons.

Taken together, over the group of 6 patients the sensitivity was ≥ 75% in 3 patients. Specificity exceeded 90% in all but the one patient who was re-operated.

### Sensitivity analysis

To motivate the choice of parameters, we tested how the sensitivity and specificity values were influenced by the energy threshold in Stage 1 of our detector on our training data set (Patient 1). Changing the threshold to 2 SD increased the maximum HFO rate to 15 1/min. However, the ranking of channels was fairly robust with respect to parameter-variations in the detector, because thresholds for EoIs are defined with respect to the variance of the data, and the recognition of HFOs depends on relative spectral properties. The combination of sensitivity/specificity for detection of the SOZ was sens  =  50% CI [13% 99%] and spec  =  91% CI [80% 97%] for thresholds of 2 SD, 2.5 SD, 3 and 4 SD. Only at 1 SD was the maximum HFO rate 30 1/min but the enhanced number of false positive HFOs and false positive HFO channels reduced specificity to 81% CI [69% 91%].

### Comparison to existing detectors

We compared the performance of our detector with two automatic detectors that are well established in literature. The first one was proposed by [11], we will refer to this detector as RMS detector. The second one was proposed by [13], we will refer to this detector as LineLength detector.

RMS detector is based on the energy defined as the moving average of the root mean square (RMS; 3-ms sliding window) amplitude of the filtered signal. Each channel was first band-pass filtered (100–500 Hz). Then segments with energy above five times the SD of the mean energy of the whole EEG during more than 6 ms were defined as HFOs. Consecutive events separated by less than 10 ms were combined as one event. Events not having a minimum of 6 peaks (band-passed signal rectified above 0 μV) greater than 3 SD from the mean baseline signal were rejected.

In LineLength detector, the short-time energy was replaced by short-time line length. The short-time line length is assumed to be less sensitive against outliers than the short-time energy. First, each channel was band-pass filtered (80–500 Hz). Then, segments with energy above five times the SD of the mean energy of the whole EEG during more than 6 ms were defined as HFOs. Consecutive events separated by less than 10 ms were combined to one event.

To benchmark RMS detector, LineLength detector and our detector with the SOZ, we then defined the HFO area by the half maximum method (section “Definition of the HFO area”). For all three detectors, the sensitivity and specificity for HFO channels to mark the SOZ is presented in Table 4. The sensitivity and the specificity were equal in 2 patients and considerably lower for RMS and LineLength detectors in 4 patients.

**Table 4:** Comparison to existing detectors.

Patient	Our detector				RMS detector				LineLength detector			
	Sens [%]	CI [%]	Spec [%]	CI [%]	Sens [%]	CI [%]	Spec [%]	CI [%]	Sens [%]	CI [%]	Spec [%]	CI [%]
1	50	13–99	91	80–97	50	13–99	91	80–97	50	13–99	94	85–99
2	14	0–64	95	84–99	0	0–40	95	84–99	0	0–40	95	84–99
3	75	19–99	94	79–99	50	7–93	97	83–100	50	7–93	94	79–99
4	75	19–99	94	84–99	0	0–60	98	90–100	25	1–81	98	90–100
5	94	70–100	39	35–87	69	41–89	52	31–73	81	54–96	61	39–80
6	13	2–40	100	86–100	13	2–40	100	86–100	13	2–40	100	86-100

We defined the HFO area by the half maximum method for all three detectors. Sens – sensitivity, spec – specificity and CI – confidence intervals as defined in section “Statistical analysis”.

### Computational aspects

We implemented the algorithm on a PC with a 64-bit operating system (Windows XP), and 8 GB RAM and 3.40 GHz CPU. For 5 minutes of recording of 50 channels with 4 kHz sampling rate the size of a file is about 60 Mbit. The computational run time for the Stage 1 (detection of EoIs) depended mainly on the choice of filters for band-pass filtering of the raw signal. Run time is dramatically decreased when using IIR filter instead of FIR family filters. For 5 min of data in a single channel, Stage 1 took 2 sec of CPU time.

The time needed in Stage 2 of the computational process depends mainly on the number of EoIs that have to be reviewed and the type of time-frequency transform. We have compared the Stockwell Transform, which was implemented in our detector, with the short-time Fourier Transform. For the 1 sec data-epoch that an EoI is reviewed, both transforms were equally fast. Recognition of an individual HFO consumes 140 ms of CPU time in our setting. However, the Stockwell Transform turned out to be superior to the short-time Fourier Transform with respect to the sharpness needed for HFO recognition in the instantaneous power spectrum (Figure 1E).

## Discussion

### Reliability of detection of the epileptogenic zone

We present here a newly developed detector for HFOs in the time-frequency domain. The final goal of the detector is to aid in rapid identification of the epileptogenic zone in order to aid in planning the surgical resection. The only proof that the epileptogenic zone has been resected is postoperative seizure freedom. In addition, healthy parts of the brain may have been resected. Because of the small number of patients and because three of the patients did not undergo surgical resection, our gold standard to approximate the epileptogenic zone is the SOZ, which was defined during presurgical workup. The SOZ provides the basis for clinical decisions. However, the definition of the SOZ may also be flawed, as can be the case in patient 5 who did not become seizure-free postoperatively and had to be re-operated on. In this patient, the HFO area exceeded the SOZ and therefore HFOs may be more sensitive markers of the epileptogenic zone.

To judge the reliability of any HFO-detector, the following assumptions must be kept in mind. 1) Based on the literature, we assume that a high rate of pathological HFOs is a reliable marker of the epileptogenic zone [4], [19], [30], [35], [36]. We assume this is also the case for patients with very different types of epilepsy. 2) However, there is not yet a decisive consensus on the exact characteristics of clinically relevant HFOs, i.e. the distinction between pathological and physiological HFOs remains uncertain [37]. We assume that our detector distinguishes pathological HFOs from artifacts and other EEG activity. We validated all detected events by visual inspection to establish their quality and veracity. 3) We distinguish between channels within or outside the HFO area and by setting a threshold in HFO rate. Other authors have used a similar approach (e.g. [9], [23]). A more elaborate approach with Kittler's method [19], [34] did not yield superior sensitivity in our data so that we propose the half maximum threshold as a simple algorithm to determine this threshold. All three of these assumptions affect the reliability of a detector.

The delineation of the SOZ is done on a channel-by-channel basis, but surgical decisions are taken in a wider context. For example, if epilepsy surgery is to be performed in form of a selective amygdala-hippocampectomy (sAHE), standard resection includes the amygdala, the anterior hippocampus proper and parts of the parahippocampal gyrus, including the entorhinal and perirhinal cortices, at least in part. If seizure recordings have lateralized the SOZ to one side and localized it within the hippocampus by recording the seizure onset with the first two contacts of a hippocampal depth electrode (HL1 and HL 2 in patient 1), it does not matter whether HFOs can be found in contact HL1, or HL2, or both of them: Both channels are usually situated within the hippocampus proper, and thus identify the same structure to be resected. Thus, our test of the HFO area against the SOZ on a channel-by-channel basis is very rigorous and leads to low values of sensitivity down to 13% (Table 3). We have chosen this test criterion because it is straightforward and common practice (e.g. [23]). A test criterion could also be based on the types of neurosurgical resection that are standard procedures in epilepsy surgery. For such a criterion, the sensitivity of our HFO detector would be higher.

### Spectral frequency of HFOs

In view of the good performance of our detector, we refrained from distinguishing HFO events into ripples and FRs, even though other authors adhere to this distinction, e.g. [11], [12], [23].

Our detector encountered events that feature their maximal power spectral density below 80 Hz. An example is depicted in Figure 4. The asymmetric distribution of HFO spectral maxima in Figure 8B suggests that HFOs may exhibit maxima even below 80 Hz. There were events, which met the requirements of EoIs, but were not recognized as HFOs because of our frequency cutoff at 60 Hz. This finding shows that the lower edge of the HFO-band is critical for HFO detection. The frequency of HFOs below 80 Hz overlaps with what is conventionally termed the gamma band [24].

### Relation between HFO and IES

It is well known that HFOs can occur simultaneously with IES [9]. By averaging over a set of 50 HFOs time-locked to an IES, it was shown that HFOs occur at frequencies distinct from those of the IES in a time-frequency plot [38]. We can reproduce this finding for individual HFOs without the need for averaging and without the need of time locking to an IES (see Figure 3 for an example).

### Advantages of the two stages of detection

We aim to distinguish HFO events from other EEG activity and artifacts. Our HFO detector involves two stages. In the first stage, possible events of interest (EoIs) are detected based on thresholds in the time domain. The sensitivity analysis for the energy threshold in our test set was presented in section “Sensitivity analysis”. In this stage of HFO detection, we emphasize sensitivity to obtain a large number of EoIs. However, high pass filtering of sharp events like IES or artifacts may cause filter ringing that is sufficiently large to be detected as “false ripples” [39].

In order to discard such events and to limit detection to distinct HFOs, we subsequently review all EoIs in the time-frequency domain. In Stage 2, our detector analyses not a fixed time window but only those time points where the high-frequency activity is above the FWHM. This enables us to look at the microstructure of the event. Conceptually this is different from methods that average over time. We computed a set of instantaneous power spectra in a time window of 1 sec. Our method relies on instantaneous power spectra as they can only be obtained by a time-frequency (TF) analysis. In our detector, no window length has to be fixed for the TF-transformation. Making use of the high time resolution, we analyzed several instantaneous spectra (5 to 50) around the Hilbert peak of each event. This detection algorithm is based on the instantaneous power spectral estimation, which periodograms or autoregressive models cannot provide at this high temporal resolution. The power spectra of this subset were not averaged either over time or over frequency, but each instantaneous power spectrum was tested with the criterion for HFO acceptance. Stage 2 of the detector thus emphasizes specificity.

The combination of these two stages provides good accuracy for detection of HFOs in human iEEG recordings.

### Comparison to existing detectors

The detection of EoI in Stage 1 of our detector was programmed along the lines of algorithms proposed by [11] and [12]. For testing purposes, we implemented RMS and LineLength detectors (section “Comparison to existing detectors”). For both detectors, the sensitivity and specificity for HFO channels was equal in 2 patients and considerably lower in 4 patients than in our detector.

The detector of [20] is, like our Stage 1, also derived from [12]. It first detects events in the time domain and then uses wavelet-based time-frequency decomposition to validate detected events visually. We went further and used the time-frequency analysis for automatic validation of detected EoIs.

Recently, a set of detectors has been described, which also detect EoIs in a first stage and then automatically recognize HFOs in a second stage in the time-frequency domain. After either Fourier Transform or Wavelet Transform, the detector proposed by [14] was shown to detect FRs in the frequency band 256–512 Hz. The choice of frequency band is restricted by decomposition levels of the dyadic wavelet transform. This method computes energy ratios in different frequency levels and has a good spectral resolution for signals in the FR frequency range. The Time-Frequency Transform by Stockwell extends the ideas of the wavelet transform such that decomposition levels can be adjusted according to the aim of detection. As a further advantage, it is based on the true frequency spectrum and globally referenced phase measurements like the Fourier transform.

The time-frequency characteristics of individual HFOs can be clearly described due to the good time-frequency resolution of the Stockwell Transform. This is an advantage over automatic or visual detection of HFOs based on data high-pass filtered at 80 Hz [3], [30]. While we also high-pass filter at 80 Hz in Stage 1 of our detector, we analyze the complete raw iEEG in Stage 2 where we review EoIs to detect HFOs.

### Limitations of the study

The thresholds of the detector were trained heuristically to optimize sensitivity/specificity in patient 1 (see section “Sensitivity analysis”). While the thresholds were kept constant for all patients in the test set, the performance of the detector should be tested with a larger group of patients.

There were events, which met the requirements of EoIs, but were not recognized as HFOs because of the frequency restriction for HFO selection. To cope with line hum, our detector is limited to events with maximal power spectral density above 60 Hz.

Furthermore, the performance is compared to the definition of the SOZ, which is the gold standard for definition of the resection margin. However, the performance of the detector should be evaluated against clinical outcome, which is beyond the scope of the current study.

### Outlook on possible clinical relevance of the detector

At its current stage of development, the detector is judged against the gold standard of the SOZ as defined by an experienced epileptologist. This is justified because the characteristics of a clinically relevant HFO are still not agreed on. Eventually the clinical relevance of the HFOs detected here should be tested against the outcome after surgery. The fact that HFOs occurred with several pathologies (Table 1) is consistent with the idea that HFOs are a biomarker of the epileptogenic zone, regardless of seizure etiology [23].

All the necessary information for HFO analysis was obtained from 5 minutes of interictal data recorded during deep sleep, at least 3 hours separated from a seizure. Contrarily, the current gold standard of presurgical diagnostics requires the recording of several epileptic seizures to delineate the SOZ. In this way, HFO analysis can potentially reduce recording time and also patient risk as it may occur due to delayed epilepsy surgery.

As an advantage, the low computational run time of the algorithm enables an online implementation of the detector. This could permit application of the detector in an intraoperative setting [40], [41]. The detector might then become a supplementary diagnostic tool for the localization of the epileptogenic zone [28].

## Conclusions

We present here a new algorithm for detection of HFOs, which was trained on one patient and tested on five other patients. The process of selecting events of interest in the time domain in a first stage focused on high sensitivity. Good specificity in HFO detection was achieved in a second stage by recognizing time-frequency characteristics of individual HFOs. Ranking channels by HFO rate and selecting only those above half maximum rate resulted in good specificity for detection of the SOZ. The sensitivity for detection of the SOZ by this algorithm was markedly higher than with RMS and LineLength detectors in four patients and equal in two patients. The computational run-time on a PC allows – in principle - an online implementation. Together with the reasonable accuracy, this holds promise for the diagnostic value of the detector for intraoperative recordings.
